# Impact of Toll-Like Receptor 2 Deficiency on Survival and Neurological Function after Cardiac Arrest: A Murine Model of Cardiopulmonary Resuscitation

**DOI:** 10.1371/journal.pone.0074944

**Published:** 2013-09-16

**Authors:** Stefan Bergt, Anne Güter, Andrea Grub, Nana-Maria Wagner, Claudia Beltschany, Sönke Langner, Andreas Wree, Steve Hildebrandt, Gabriele Nöldge-Schomburg, Brigitte Vollmar, Jan P. Roesner

**Affiliations:** 1 Clinic for Anaesthesiology and Critical Care Medicine, University Hospital Rostock, Rostock, Germany; 2 Institute for Neuroradiology, University Hospital Greifswald Greifswald, Germany; 3 Department of Anatomy, Rostock University, Rostock, Germany; 4 Institute for Experimental Surgery, Rostock University, Rostock, Germany; University of Tübingen, Germany

## Abstract

**Background:**

Cardiac arrest (CA) followed by cardiopulmonary resuscitation (CPR) is associated with poor survival rate and neurofunctional outcome. Toll-like receptor 2 (TLR2) plays an important role in conditions of sterile inflammation such as reperfusion injury. Recent data demonstrated beneficial effects of the administration of TLR2-blocking antibodies in ischemia/reperfusion injury. In this study we investigated the role of TLR2 for survival and neurofunctional outcome after CA/CPR in mice.

**Methods:**

Female TLR2-deficient (TLR2^-/-^) and wild type (WT) mice were subjected to CA for eight min induced by intravenous injection of potassium chloride and CPR by external chest compression. Upon the beginning of CPR, n = 15 WT mice received 5 µg/g T2.5 TLR2 inhibiting antibody intravenously while n = 30 TLR2^-/-^ and n = 31 WT controls were subjected to injection of normal saline. Survival and neurological outcome were evaluated during a 28-day follow up period. Basic neurological function, balance, coordination and overall motor function as well as spatial learning and memory were investigated, respectively. In a separate set of experiments, six mice per group were analysed for cytokine and corticosterone serum levels eight hours after CA/CPR.

**Results:**

TLR2 deficiency and treatment with a TLR2 blocking antibody were associated with increased survival (77% and 80% vs. 51% of WT control; both *P < 0.05*). Neurofunctional performance was less compromised in TLR2^-/-^ and antibody treated mice. Compared to WT and antibody treated mice, TLR2^-/-^ mice exhibited reduced IL-6 (both *P < 0.05*) but not IL-1β levels and increased corticosterone plasma concentrations (both *P < 0.05*).

**Conclusion:**

Deficiency or functional blockade of TLR2 is associated with increased survival and improved neurofunctional outcome in a mouse model of CA/CPR. Thus, TLR2 inhibition could provide a novel therapeutic approach for reducing mortality and morbidity after cardiac arrest and cardiopulmonary resuscitation.

## Introduction

Despite many years of laboratory and clinical research, survival rate and neurofunctional outcome following cardiac arrest (CA) and cardiopulmonary resuscitation (CPR) remain poor. The incidence of cardiac arrest in Europe is 38 per 100.000 and only 10.7% of patients survive until discharge from hospital [1]. Of all patients with return of spontaneous circulation (ROSC), 80% are comatose and more than 70% are unable to return to an independent way of life due to neurological deficits [2]. Most patients admitted to the intensive care unit following an initially successful CPR develop post-resuscitation disease with serious neurological impairment and multiple organ dysfunction syndrome [3]. The underlying mechanisms include myocardial dysfunction, endothelial damage [4], leukocyte activation and increased leukocyte-endothelial interaction [5]. Regarding the recovery of these patients, the most limiting factor is the poor neurological outcome. Over the past decades, only few strategies have proven effective to limit post-ischemic injury such as controlled hypothermia and symptomatic treatment of accompanying complications [6].

Toll-like receptors play a key role in innate immune responses by recognising components of microbial organisms and initiating inflammatory cascades [7]. In addition to microbial pathogens, TLR2 can also be activated by non-microbial ligands predominantly liberated by inflamed or necrotic tissues. There is evidence that TLR2 activation is associated with organ dysfunction under pathological conditions such as traumatic haemorrhage and reperfusion injury [8]. The inhibition of TLR2 pathways has been shown to be associated with beneficial effects from reperfusion injury to the heart [9], kidney [10] and brain [11]. However, no data exist on inhibition of TLR2 pathways regarding survival and neurofunctional outcome after CA/CPR so far.

In a murine model of CA/CPR we aimed to investigate the effects of TLR2 deficiency or treatment with a TLR2 blocking antibody on survival and neurological function in a 28-day post-resuscitation observation period.

## Materials and Methods

All animal procedures were in accordance with institutional, national and European guidelines for the care and use of laboratory animals (European Communities Council Directive of November 24^th^, 1986 [86/609/EEC]). The protocol was approved by the Ethical Committee of the Landesamt für Landwirtschaft, Lebensmittelsicherheit und Fischerei, Mecklenburg-Vorpommern, Germany (Permit Number: LALLF M-V/TSD/7221.3-1.1-073/10). All efforts were made to minimize suffering.

### Cardiac Arrest and Cardiopulmonary Resuscitation

Female wild type (WT, C57BL/6NCrl code 027; n = 31) and TLR2-deficient (TLR2^-/-^, B6.129-Tlr2 ^tm1Kir^/J; n = 30) mice at 12-16 weeks of age exhibiting 20-24 g body weight were anaesthetised by intraperitoneal (i.p.) injection of 12 µg/g ketamine + 8 µg/g xylazine and subjected to oral intubation employing a 22-gauge intubation cannula. Mechanical ventilation was initiated with a FiO_2_ (fraction of inspired oxygen) of 0.4, a tidal volume of 10 µL/g and a respiratory rate of 130 breaths per minute (MiniVent; Hugo Sachs, March, Germany). Mice were placed on an autoregulated heating plate and body temperature was continuously monitored by a rectal thermocouple probe (Effenberger, Pfaffingen, Germany). A polyethylene catheter (PE 50, ID 0.28 mm; Portex, Hythe, UK) was inserted into the right jugular vein and blood pressure was monitored by a non-invasive blood pressure device (A D Instruments, Spechbach, Germany). Needle probe electrocardiogram (ECG) monitoring was initiated and blood pressure and ECG were recorded employing PC-based data acquisition (LabChart 5 pro, ADInstruments, NSW, Australia).

Cardiac arrest (CA) was induced by injection of 80 µg/g potassium chloride and mechanical ventilation was interrupted upon verification of cardiac arrest by ECG. Resuscitation was initiated following eight min of CA, ventilation was resumed (220/min; FiO_2_ 1.0), precordial chest compressions were begun with a frequency of 450/min employing a modified sewing machine as previously described [12] and 0.4 µg/g epinephrine were injected. At the beginning of CPR, a subset of WT mice (n = 15) were subjected to treatment with 5 µg/g of T2.5 TLR2-inhibiting antibody (Hycult Biotech, The Netherlands) dissolved in 200 µL saline via the jugular vein catheter. All WT controls as well as TLR2-deficient mice received 200 µL of isotonic saline serving as vehicle control. Following two min of CPR, ventilation was reduced to 130/min, FiO_2_ 0.6 and returned to baseline (FiO_2_ 0.4) after 20 min of CPR. One hour after the return of spontaneous circulation (ROSC), the jugular vein catheter was removed, wounds were surgically ligated and mice were weaned from mechanical ventilation. ECG probes, blood pressure device and rectal thermocouple probe were removed immediately prior to extubation. To prevent dehydration, mice received 0.5 mL of isotonic saline (s.c.) before being returned to their cages. After having been returned to their cages, resuscitated mice had free access to food and water. Within the first 24 h, metamizol (2.5 mg/mL) was added to the water to manage pain resulting from surgical procedures.

All mice were weighed on the day of CPR (d 0, prior CA/CPR) as well as on each of the following 14 days and the day of the end of the observation period of the study (d 28).

All resuscitated mice were closely monitored during the observation period of 28 days (between 8 a.m. to 8 p.m. every 2 hours, at night every 4 hours) for survival and physiological conditions such as breathing pattern, spontaneous movements, the ability to ingest food and grooming.

In accordance with the guidelines for animal experiments, a loss of body weight of more than 30% compared to baseline value would lead to the decision to remove the animal from the experiment by sacrifice due to an overdose of i.p. injected ketamine/xylazine. In the present study, no resuscitated mice met this criterion.

### Analysis of Neurological Function

#### NeuroScore

Scoring for neurological evaluation was performed employing a modified grading system for mice comprising the following items: Level of consciousness, corneal reflex, respirations, righting reflex, coordination and movement/activity [13,14]. Within each item, 0, 1 or 2 points were achievable resulting in 12 points representing the maximum score. Assessment was performed by an unbiased observer.

#### RotaRod test

The RotaRod paradigm is a test to study motor function, balance and coordination [15,16]. Mice were subjected to balancing on a rotating cylinder (12.5 revolutions/min) for three attempts of 300 sec (900 sec in total) and the time until mice fell off the rod was recorded. Both NeuroScore and RotaRod test were applied on the day of CA/CPR (d 0, 1 h prior to CA/CPR) as well as on each of the following days until day 5 and on day 7, 14 and 28 after CA/CPR.

#### Water Maze test

The Water Maze test was employed for the investigation of spatial learning and memory of laboratory animals [17]. Mice were trained daily (twice a day at 8 am and 6 pm, five attempts in each session) beginning on day 5 until the day before CA/CPR to find a 5 x 5 cm escape platform located 0.5 cm below the water surface in a circular tank (60 cm in diameter, 40 cm in height) filled with water. On the third day of training, visibility of the platform was prevented by the addition of milk to the water. For the investigation of mice memory function following CA/CPR, the time required to find the platform was again measured when animals were placed at the same starting position within the tank. In order to avoid loss of animals from drowning due to general weakness or catabolic state, mice underwent the test after CA/CPR only when they a) had reached a maximum score in the NeuroScore; b) had fulfilled an attempt of 300 sec on the RotaRod and c) exhibited a halt in loss of body weight (day X following CA/CPR). The test was then performed daily until day X+5.

### Analysis of Cytokine and Corticosterone Plasma Levels

An additional n = 6 mice per group were subjected to blood withdrawal two weeks before and eight hours following CPR by median laparotomy and puncture of the inferior caval vein. Plasma samples were stored at -80°C pending analysis. Corticosterone (Demeditec Diagnostic GmbH, Kiel, Germany), interleukin-1β and interleukin-6 (Thermo Scientific, Pierce Biotechnology, Rockford, USA) were determined employing enzyme-linked immunosorbent assay (ELISA) and a microplate reader (Sunrise Remote, TECAN Austria GmbH, Salzburg, Austria).

### Statistical Analysis

Statistical analysis was performed employing SigmaPlot 10 (Jandel Corporation, San Rafael, CA, USA) and statistical significance was defined as *P < 0.05*. Data for Kaplan-Meier Survival Analysis were tested with the log-rank test. For multiple comparison analysis Holm-Sidak method was used. For analysis of quantitative data, either student’s t-test (if normal distribution applied) or Mann-Whitney rank sum test was employed and results are expressed as mean ± standard deviation (SD) or as median and [25-75] percentiles, respectively. For multiple comparisons ANOVA-test with Bonferroni adjustment was performed. A numeric difference in participants on Water Maze test after CA/CPR was evaluated by Chi-Square test.

## Results

### TLR2-deficiency and functional blockade is associated with increased survival after CA/CPR

Following eight min of CA, all mice exhibited return of spontaneous circulation (ROSC) following CPR and epinephrine injection and were successfully weaned from mechanical ventilation. There were no significant differences in duration of CPR, dosage of epinephrine, body temperature, mean arterial pressure and heart rate between the groups. As an indication for a faster recovery after CA/CPR the time needed for weaning from mechanical ventilation was significantly reduced in TLR2^-/-^ mice and antibody treated mice (WT + T2.5) compared to WT controls (Table 1).

**Table 1:** Global parameters of CA/CPR.

	**WT**	**TLR2^-/-^**	**WT + T2.5**
	n = 31	n = 30	n = 15
**baselinebeforepreparationforCA/CPR**			
body weight [g]	22,3 ± 1,4	22,1 ± 0,6	21,2 ± 0,8
heart rate [1/min]	218 ± 24	229 ± 40	224 ± 21
mean arterial pressure [mm Hg]	95 ± 13	102 ± 13	97 ± 17
body temperature [°C]	36,1 ± 0,3	36,2 ± 0,1	36,3 ± 0,1
**parameterofCA/CPR**			
time ROSC [sec]	80 ± 15	73 ± 20	66 ± 26
successfully resuscitated animals [%]	100	100	100
dosage of epinephrine [µg]	10 [10-20]	10 [10-15]	10 [10-15]
weaning from mech. ventilation [min]	150 ± 20	128 ± 17*	113 ± 20*
**1hourafterCA/CPR**			
heart rate [1/min]	401 ± 101	358 ± 75	365 ± 80
mean arterial pressure [mm Hg]	83 ± 21	75 ± 17	80 ± 25
body temperature [°C]	36,1 ± 0,1	36,0 ± 0,3	36,1 ± 0,2

Analysis of global parameters of CA/CPR. There were no significant differences between the groups, except the time needed for weaning from mechanical ventilation.

Data are presented as median and [25-75] percentiles and were analysed employing ANOVA/Bonferroni. *P < 0.05 WT vs. TLR2^-/-^; #*P* < 0.05 WT vs. WT+T2.5

**Figure 1:**
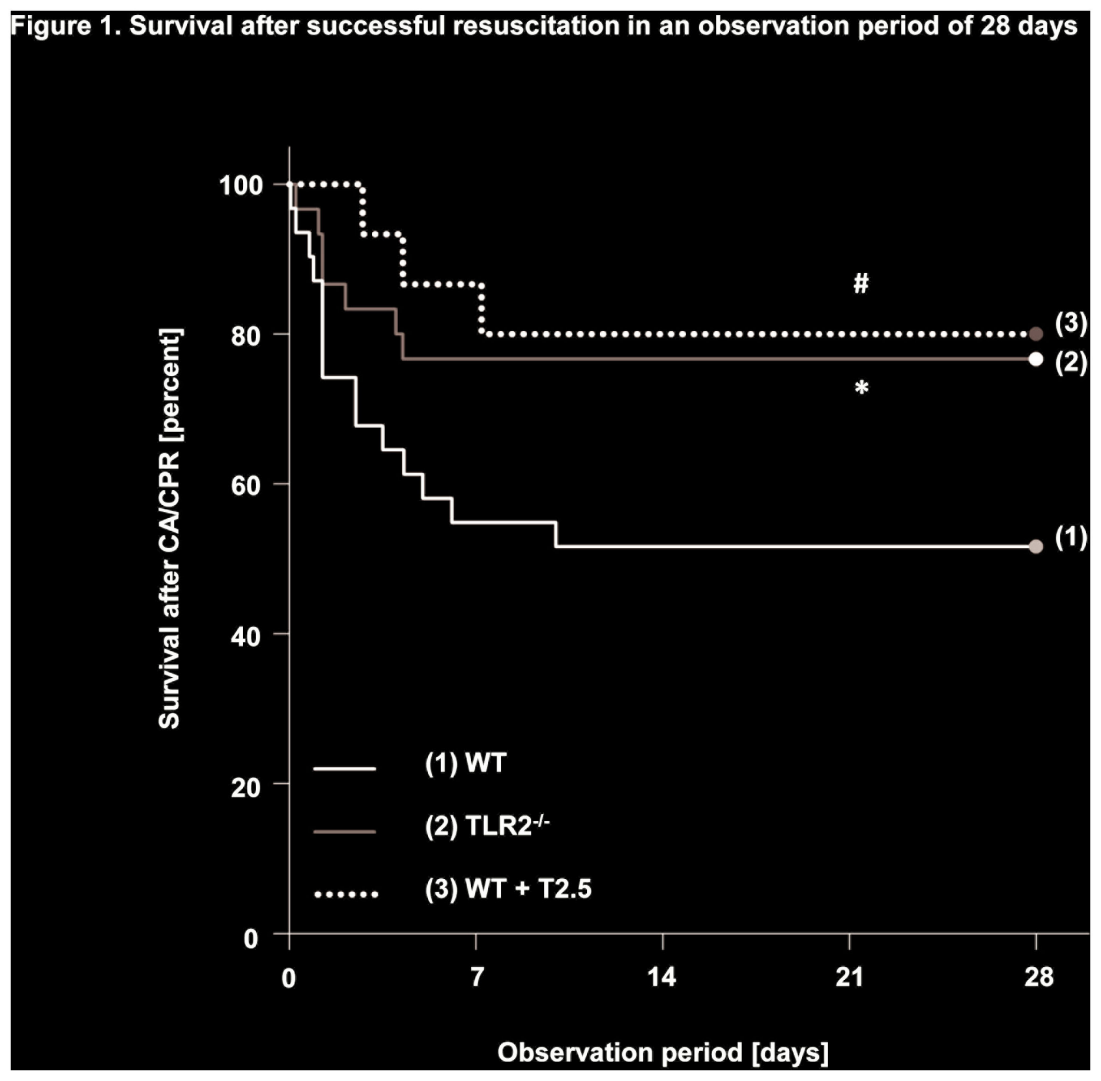
Survival after successful resuscitation in an observation period of 28 days. Survival of mice following eight min of cardiac arrest (CA) and cardiopulmonary resuscitation (CPR) within the 28-day observation period. Black line (1): wildtype (WT) controls (n = 31), gray line (2): TLR2-deficient (TLR2^-/-^, n = 30), dotted line (3): antibody (T2.5)-treated WT mice (n = 15). Data was analysed by Kaplan-Meier log-rank survival analysis and pairwise multiple comparison procedures (Holm-Sidak method). *P < 0.05 WT vs. TLR2^-/-^; #*P < 0.05* WT vs. WT+T2.5.

**Figure 2:**
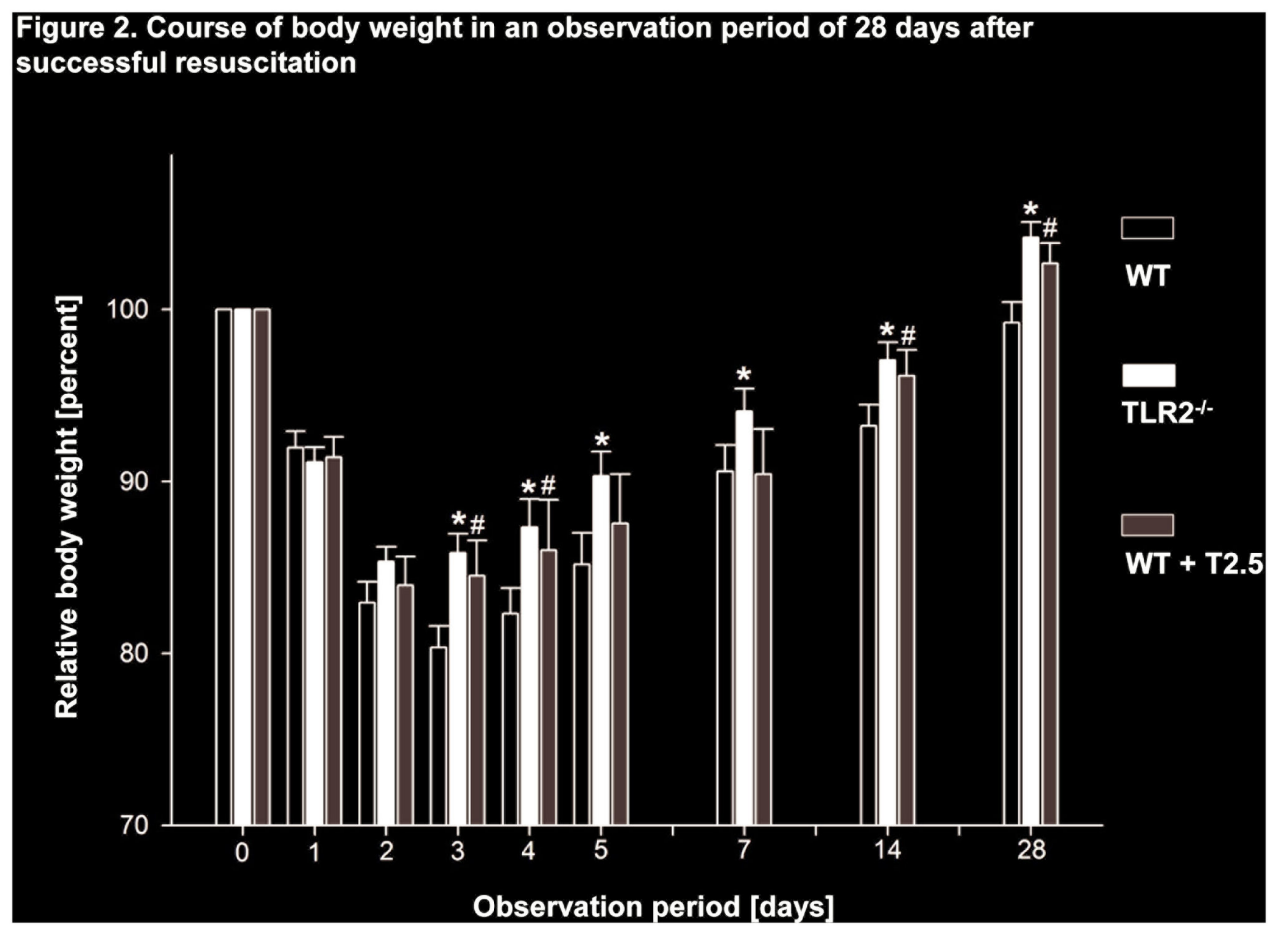
Course of body weight in an observation period of 28 days after successful resuscitation. Course of mouse body weight before (day 0) and following CA/CPR within the 28-day observation period displayed as relative body weight to compared to day 0. Data is presented as mean ± SD and was analysed by One-way ANOVA followed by Bonferroni test for multiple comparisons. White columns WT, black columns TLR2^-/-^, grey columns WT+T2.5. *P < 0.05 WT vs. TLR2^-/-^; #*P < 0.05* WT vs. WT+T2.5.

During the 28 day observation period of the study, TLR2^-/-^and WT mice subjected to treatment with a TLR2-inhibiting antibody showed increased survival compared to WT controls (77% and 80% vs. 51%; *P < 0.05*). Mortality was highest within the first 10 days after CA/CPR in all groups and declined thereafter (Figure 1). In line with these results, both TLR2^-/-^ and antibody-treated WT mice exhibited a reduction in loss of body weight beginning on day 3 after CA/CPR compared to WT controls (85.8 ± 1.2 and 84.3 ± 1.8 vs. 80.4 ± 1.3% of original body weight prior to CA/CPR; *P < 0.05*) and this difference persisted until day 28 (Figure 2). Moreover and in contrast to WT controls, both TLR2^-/-^ and antibody-treated WT mice regained weight above the level of their original body weight throughout the observation period.

### TLR2^-/-^ and antibody-treated WT mice exhibit improved neurofunctional outcome

**Figure 3:**
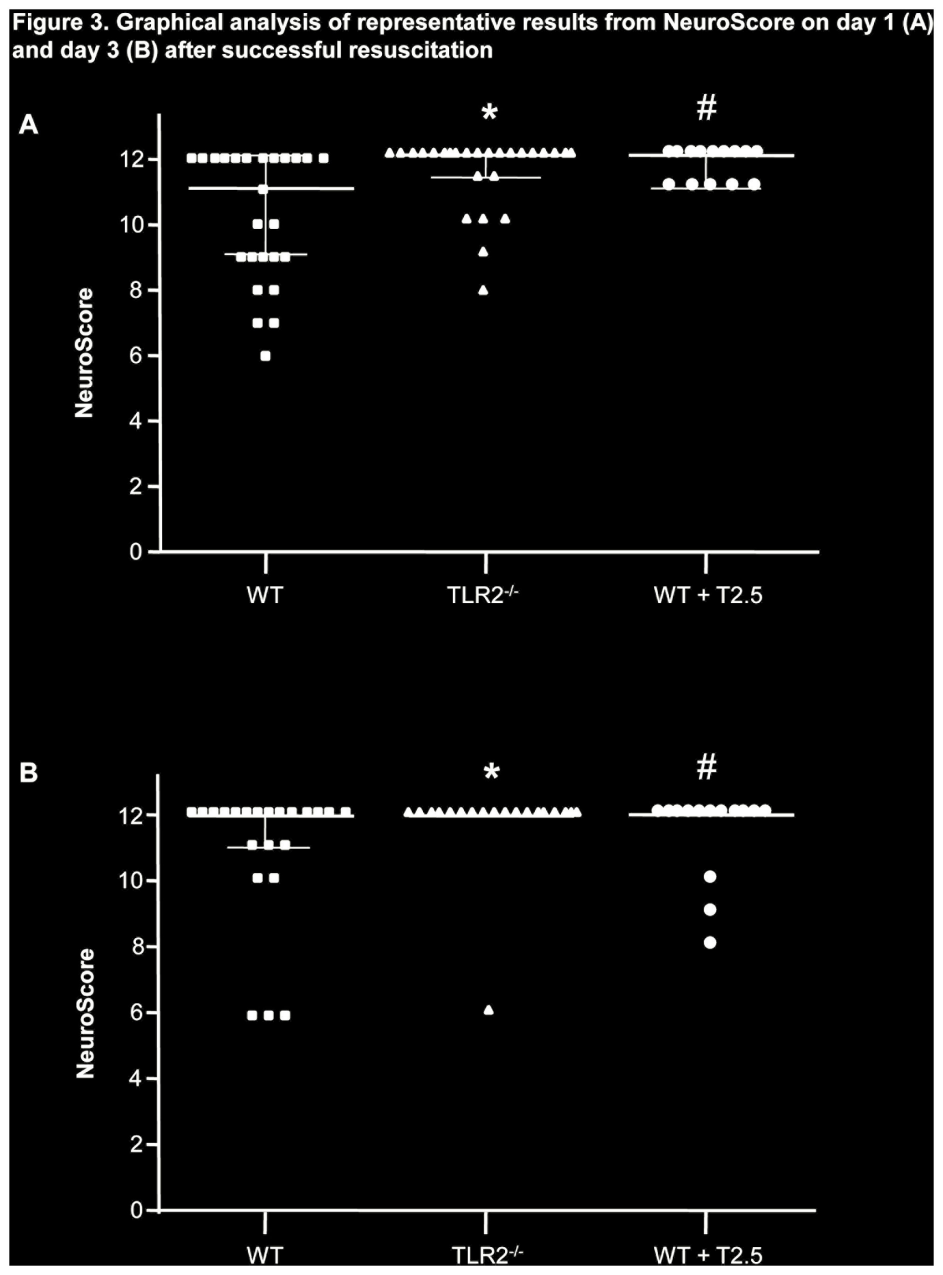
Graphical analysis of representative results from NeuroScore. Representative results from evaluation of mice on day 1 (A) and day 3 (B) after CA/CPR employing the NeuroScore. Data are presented as median and interquartile range and was analysed employing ANOVA/Bonferroni. *P < 0.05 WT vs. TLR2^-/-^; #*P < 0.05* WT vs. WT+T2.5.

After resuscitation, daily evaluation employing the NeuroScore revealed reduced neurofunctional impairment in TLR2^-/-^ and antibody-treated WT mice compared to WT controls (*P < 0.05* for the comparison of TLR2^-/-^ on day 1 through 3 and of antibody-treated animals on day 1 and 3 compared to WT controls; graphical plotting of NeuroScore evaluation on day 1 and day 3 Figure 3). Similar results were obtained employing the RotaRod test (Table 2). In contrast to WT controls, TLR2^-/-^ mice showed no impairment in their ability to balance on the rotating cylinder on the first day after CA/CPR (3 completely fulfilled attempts of 300 sec [i.e. 900 sec] in median compared to a total of 74.5 sec in median within 3 attempts of WT controls; *P < 0.05*). Antibody-treated WT mice exhibited an only mild shortening of the time on the rod on day 1 after CA/CPR (629 sec in median; *P < 0.05* vs. WT control) that returned to the level of performance prior to resuscitation (900 sec in median on day 5). WT controls however did not regain their ability to balance on the rod for a total of 900 sec but continued to exhibit impaired performance until 5 days after CA/CPR (667 sec in median, Table 2). From day 7 until the end of the observation period on day 28 after CA/CPR, differences between groups were no longer detectable (data not shown).

**Table 2:** Results and Analysis of RotaRod Test.

**RotaRod**	**WT**	**TLR2^-/-^**	**WT + T2.5**
(time [sec])	n = 31	n = 30	n = 15
day 0	900 [723-900]	900 [837-900]	900 [845-900]
day 1	75 [9-445]	900 [697-900]*	629 [155-853]^#^
day 2	292 [58-453]	900 [900-900] *	580 [457-900]^#^
day 3	380 [93-638]	900 [807-900]*	488 [488-900]
day 5	667 [356-900]	900 [837-900]*	900 [433-900]

Results from neurological evaluation employing the RotaRod Test before (day 0) and on day 1-3 and day 5 after CA/CPR. Data is presented as median and [25-75] percentiles and was analysed employing ANOVA/Bonferroni. *P < 0.05 WT vs. TLR2^-/-^; #*P* < 0.05 WT vs. WT+T2.5

The time for participation in the Water Maze test after successful resuscitation serves as an indicator for overall neurofunctional recovery. Regarding the requirements for participation in the Water Maze test (physical status and performance in NeuroScore and RotaRod test), TLR2^-/-^ mice and the antibody-treated WT mice met the criteria two days earlier compared to WT controls (day X defined as the first day after CA/CPR mice were subjected to the test; 5 [3-11] days vs. 3 [3-6] and 3 [3,4] days, respectively; both *P < 0.05*). Considering increased mortality among WT controls, also higher numbers of TLR2^-/-^ and antibody-treated WT mice could be subjected to further cognitive evaluation (76.6% and 80% vs. 51.6%; both *P < 0.05*; Table 3). During the daily training phase of the Water Maze test from day 5 until 1 day prior to CA/CPR, all WT mice showed a rapid decline in the time required to find the hidden platform (times for first and last attempt prior to CA/CPR are exemplarily shown in Table 3). Following CA/CPR, both control- and antibody-treated WT mice remembered the location of the hidden platform indicated by comparable times needed to escape from swimming in the tank. However, WT control animals were not able to further reduce the time being forced to swim 5 days later. In contrast, T2.5 antibody-treated WT mice could further minimize the time needed to rescue themselves onto the escape platform (day X: 8 [7-30] sec vs. day X+5: 2 [2-7] sec, *P < 0.05*), indicating a more intact ability of learning and memory function compared to WT controls (day X+5: 6 [3-11] sec; *P < 0.05* vs. antibody-treated WT controls). An interesting observation was made in TLR2-deficient mice. In contrast to WT mice, TLR2^-/-^ animals did not show any signs of learning as they were not able to reduce the time needed to find the hidden platform during the training phase (16 [9-39] sec at their second attempt compared to 14 [9-19] sec at their first). Following CA/CPR, no further reduction in the time required for self-rescue was observed. These findings may be associated with previous findings of impaired hippocampal neurogenesis in TLR2^-/-^ mice [18].

**Table 3:** Results and Analysis of spatial learning employing Water Maze Test.

**WaterMazeTest**	**WT**	**WT + T2.5**	**TLR2^-/-^**
	n = 31	n = 15	n = 30
**TrainingphasebeforeCA/CPR** (time [sec])			
first attempt (day -5)	13 [8-38]		14 [9-19]
last attempt (day -1)	9 [3-23]*		16 [9-39]
**ParticipationintheWaterMazeTestafterCA/CPR**			
participants after CA/CPR			
n [%]	16/31 [52]	12/15 [80]^#^	23/30 [77]^&^
earliest day of			
participation after CA/CPR	5 [3-11]	3 [3-4]^#^	3 [3-6]^&^
(day X)			
**TestafterCA/CPR** (time [sec])			
first attempt (day X)	9 [4-21]	8 [7-30]	16 [7-23]
last attempt (day X + 5)	6 [3-11]	2 [2-7]^§^	16 [6-31]

Results from neurological evaluation employing the Water Maze Test. Mice were trained daily (twice a day, five attempts in each session) beginning 5 days prior to CA/CPR; results from the first and last attempt of the training phase are shown in the upper third of the table. *P < 0.05 WT day - 5 vs. day - 1 Participation of mice after CA/CPR (defined as day X) was restricted to animals that met the requirements for participation (i.e. 12 points in the NeuroScore, a completed attempt in the RotaRod Test (300 s), halt in loss of body weight and no signs of disturbance). Number and earliest time point of participation of mice in the Water Maze Test following CA/CPR is presented in the middle part of the table. #*P* < 0.05 WT vs. WT+T2.5; & *P < 0.05* WT vs. TLR2^-/-^. First and last attempt of mice after CA/CPR is presented in the lower third of the table. §*P* < 0.05 WT+T2.5 day X vs. WT+T2.5 day X+5. Data is presented as median and [25-75] percentiles and was analysed employing ANOVA/Bonferroni and Chi-Square test for participants after CA/CPR.

### TLR2^-/-^ mice showed alterations in corticosterone and cytokine levels

Eight hours after CA/CPR, increased corticosterone plasma levels were detected in all groups of mice possibly reflecting an ubiquitous stress response resulting from CA/CPR (Table 4). However, in TLR2^-/-^ mice corticosterone concentrations were higher compared to WT and antibody-treated WT mice (1269 ± 66 vs. 932 ± 125 and 801 ± 95 nmol/l, respectively; *P < 0.05*). In contrast, TLR2^-/-^mice showed a tendency towards a reduced increase in interleukin-1β (IL-1β) plasma levels (0.42 ± 0.28 vs. 0.72 ± 0.28 and 0.88 ± 0.21 pg/mL in WT and antibody-treated WT mice, respectively) and interleukin-6 (IL-6) concentrations eight hours following CA/CPR (237 ± 48 vs. 1949 ± 449 and 2167 ± 307 pg/mL in WT and antibody-treated WT mice, respectively; both *P < 0.05* vs. TLR2^-/-^). Therefore, evaluation of mice response to CA/CPR with regard to stress hormones and inflammatory mediators did not reveal a correlation to the observed differences in increased survival and improved neurological function but appeared to be determined by mice genotype.

**Table 4:** Results of inflammatory response after cardiac arrest and resuscitation.

	**WT**		**TLR2^-/-^**		**WT + T2.5 antibody**	
	n = 6		n = 6		n = 6	
	baseline	8 h after CA/CPR	baseline	8 h after CA/CPR	baseline	8 h after CA/CPR
Corticosteron [nmol/L]	96 ± 3	932 ± 125	98 ± 3	1269 ± 66 *^#^	95 ± 3	801 ± 95
Interleukin-1β [pg/mL]	0.5 ± 0.1	0.7 ± 0.3	0.2 ± 0.1	0.4 ± 0.3	0.4 ± 0.1	0.9 ± 0.2
Interleukin-6 [pg/mL]	18 ± 11	1949 ± 449	46 ± 13	237 ± 48 *^#^	22 ± 9	2167 ± 307

Plasma levels of corticosterone and interleukin-1β and interleukin-6 (IL-1β and IL-6) at baseline and eight hours following CA/CPR. Data is presented as mean ± SD and was analysed by ANOVA/Bonferroni. Eight hours after CA/CPR: *P < 0.05 WT vs. TLR2^-/-^; #*P* < 0.05 WT vs. WT+T2.5

## Discussion

In the present study, we investigated the effects of TLR2 pharmacological blockade by an inhibiting antibody as well as genetic TLR2 deficiency on survival and neurofunctional outcome after cardiac arrest (CA) and cardiopulmonary resuscitation (CPR) in mice. Employing a modified sewing machine, the experimental setup employed in the study allows for highly standardised performance of CPR with regard to frequency and depth of chest compressions [19]. As the study aimed to dissect the role of TLR2 in post-resuscitation disease, a continuing process extending up to weeks after CPR, a long-term observation period (i.e. 28 days) was chosen to investigate outcome and neurofunctional performance in a context of clinical relevance. Taken together, we provide evidence that both inhibition and absence of TLR2 signalling is associated with reduced mortality, improved locomotive function and enhanced preservation of higher cognitive capacity following CA/CPR.

Although TLR2 has been implicated in the pathogenesis of ischemia- and reperfusion-injury [20], the role of TLR2 in post-resuscitation disease has not been investigated so far. In sterile inflammatory conditions, TLR2 activation is mediated by damage associated molecular patterns (DAMPs) such as oxygen radicals, HMGB1, fibrinogen and arachidonic acid [21]. DAMPs are liberated from host tissues upon cellular stress and TLRs support the activation of further pro-inflammatory signal transduction cascades. Hypoxia induces TLR2 expression in neuronal tissue [22,23] and TLR2 deficiency or inhibition by function blocking antibodies has been shown to reduce the transcription of inflammatory genes, inflammatory cell-infiltration and neuronal cell death following focal cerebral ischemia [11,24,25]. In the present study, histological analysis of animals that had died within the first hours or days following CA/CPR revealed apoptotic cells in hippocampal cerebral tissue of WT mice (Figure S1). However, these findings were not clearly correlated to neurofunctional outcome or mortality of WT mice following CA/CPR as no signs of neurological damage could be detected in other WT mice that had as well died shortly after CA/CPR. In this regard however, histological signs of ischemic hepatitis, pulmonary oedema und pulmonary failure were frequently encountered (Figure S2), suggesting diverse pathophysiological causality for neurofunctional impairment or death within the first days after cardiac arrest and resuscitation. As the main aim of the present study was to focus on long-term effects and neurofunctional outcome following CA/CPR, resuscitated animals were not subjected to euthanasia and systematic histological analysis shortly after CA/CPR. TLR2 is expressed on vascular endothelial cells, circulating inflammatory cells and a variety of other tissues and we may only speculate on the contribution of TLR2 deficiency and antibody treatment on a reduction of inflammation-mediated damage to neuronal tissue, a general preservation of vascular barrier function or improved organ function that in turn promotes cerebral tissue integrity following CA/CPR. Given this limitation of the present study, further studies are needed to identify the underlying pathophysiological cause of the beneficial effects of TLR2 deficiency and blockade on survival and neurofunction after CA/CPR.

Evaluation of neurofunctional basal parameters determined by NeuroScore within the first days following CA/CPR revealed statistically significant improved performance of TLR2^-/-^ and antibody-treated WT mice compared to WT controls. TLR2 is expressed in neurons, glia cells and astroytes. Okun et al. speculated for a role of TLR2 in cognition and showed that TLR2 profoundly alters neuronal cell proliferation and differentiation [26]. TLR2^-/-^ mice were not compromised in the RotaRod Test compared to WT mice and antibody-treated WT mice showed an only minimal degree of impairment. The results obtained in the Water Maze Test investigating spatial learning and memory further supported the results of a protection of mice from TLR2 pharmacological inhibition but not those related to TLR2 genetic deficiency, since TLR2^-/-^ mice did not show any degree of learning in the training phase. In this regard, absence of TLR2 has been associated with impaired hippocampal neurogenesis [18], the cerebral region primarily responsible for learning and memory and TLR2^-/-^ mice may therefore not be suitable candidates for the evaluation of these cognitive functions. Since mice were subjected to the Water Maze Test only if they had met the requirements for body weight, NeuroScore and performance in the RotaRod Test, the results of the evaluation of learning and memory have to be interpreted with respect to a positive-selection of animals. However, the significant earlier time point of admission to the Water Maze test of both TLR2^-/-^ and antibody-treated WT mice further supports our result of a benefit of these animals compared to WT controls.

The production of both stress hormones and inflammatory cytokines has been shown to be affected by TLR2 [27,28]. In the present study, TLR2-deficiency was accompanied by reduced plasma levels of inflammatory mediators after CA/CPR. Although similar findings of a diminished inflammatory response have been associated with survival following CA/CPR also in humans [29], TLR2-inhibiting antibody-treated WT mice exhibiting comparable outcome after CA/CPR as TLR2^-/-^ mice showed increased IL-1β and IL-6 levels comparable to WT controls. Thus, the benefits from TLR2 inhibition or deficiency may not be attributable to reduced levels of post-resuscitation-related inflammatory mediators alone. In addition, stress hormone (i.e. corticosterone) plasma levels were highest in TLR2^-/-^ mice and we hypothesize that these findings may further support the notion of a general reduction in inflammatory response following the CA/CPR trauma. On the other side the use of hydrocortisone with the aim to reduce the inflammatory response nonspecifically failed to show any beneficial effects in treatment of patients suffering from post-cardiac arrest syndrome [30,31]. A recent study in a murine model of cardiac arrest and resuscitation revealed beneficial effects on neuroinflammation and neuronal death by specific processes due to the cholinergic anti-inflammatory pathway and the involved cholinergic α7 nicotinic receptors [32].

We suggest that other but also specific TLR2-related mechanisms are responsible for the beneficial effects of TLR2-deficiency in our animal model of cardiac arrest and resuscitation, however further studies are needed to resolve these mechanisms.

Body weight of the laboratory mouse is a sensitive parameter of general wellbeing [33]. WT control animals exhibited prolonged loss of body weight following CA/CPR and showed full recovery of body weight not before the end of the observation period on day 28. In contrast, increased survival and neurofunctional performance in both TLR2^-/-^ and antibody-treated WT mice was associated with a rapid halt in loss of body weight and weight gain above the level prior to CA/CPR. During autopsies and histological analysis of the primarily WT control animals dying within the first 10 days after CA/CPR, we found a variety of pathologies such as pulmonary edema and leukocyte infiltration, ischemic hepatitis and acute renal failure. Interestingly, during the first stage of model development, primarily surviving male mice subjected to the experiments presented with urinary outflow obstruction and subsequent post-renal failure following death on day 4 through 10 after CA/CPR, as has been proven by autopsies. Therefore, the present study was conducted employing female mice only and no such pathology had been observed in these mice.

## Conclusion

Deficiency of TLR2 and TLR2 inhibition by a blocking antibody resulted in an increased survival and improved neurofunctional outcome in a mouse model of CA/CPR. The mode of protection appears to be independent from a reduction of inflammatory cytokines. Although the explanatory power of neurofunctional outcome in TLR2 deficient animals may be limited due to general differences in innate cerebral architecture compared to WT mice, they do not affect the evidence for the beneficial effects of anti-TLR2 antibody treatment for neurofunctional outcome after CA/CPR. Further studies are necessary to elucidate the underlying mechanism and subsequently to further establish TLR2 inhibition as a potential innovative therapeutic approach of reducing mortality and morbidity after cardiac arrest and cardiopulmonary resuscitation.

## Supporting Information

Figure S1 **Cardiac arrest induced neuronal tissue alterations.** Histological sections employing Nissl´ staining from a healthy control mouse (**B**) and a resuscitated animal five days after CA/CPR (**D**) showing the hippocampus (left panels) and a magnification of the hippocampal CA1/CA-2 region (right panels). Following CA/CPR, pyramidal cells of the CA-1 region show signs of neuronal cell apoptosis (right panel, D). (**C**) and (**D**) are findings in the same animal that suggest an excellent correlation between NMR and histological examinations.
(TIF)

Figure S2 **Ischemic hepatitis and pulmonary failure 5 days after successful resuscitation.** Histological sections of the liver of a WT mouse five days after CA/CPR, signs of ischemic hepatitis and apoptotic cells are visible in hematoxylin eosin (HE) (**A**) and leukocyte infiltration in chloroacetate esterase (CAE) staining (**B**). **C** Histological sections of a WT mouse lung five days after CA/CPR exhibiting pulmonary oedema and alveolar membrane thickening in HE and **D** leukocyte infiltration in CAE staining. 100x and 200x indicate magnification.
(TIF)

Checklist S1 **ARRIVE guidelines.** Checklist for the experimental setup based on the ARRIVE-guidelines (Animals in Research: Reporting in vivo Experiments) Introduction: “Impact of Toll-like receptor 2 deficiency on survival and neurological function after cardiac arrest: A murine model of cardiopulmonary resuscitation”.
(DOCX)
