# Supplementary figures and images for: Impact of Toll-Like Receptor 2 Deficiency on Survival and Neurological Function after Cardiac Arrest: A Murine Model of Cardiopulmonary Resuscitation
Source: PLoS One. 2013 Sep 16;8(9):e74944. doi: 10.1371/journal.pone.0074944 (PMC3774715; doi:10.1371/journal.pone.0074944)

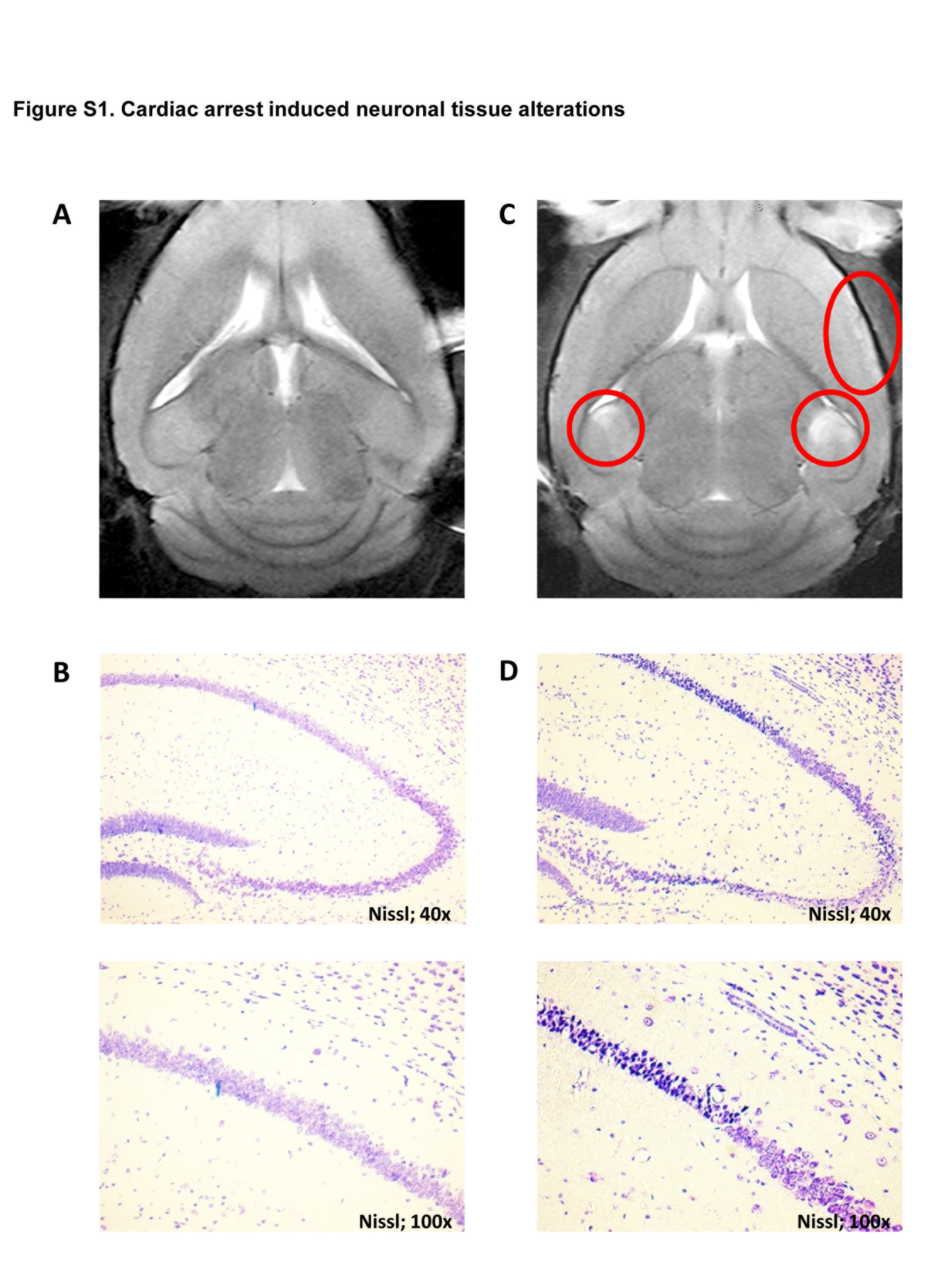

Supplement: Figure S1 — Cardiac arrest induced neuronal tissue alterations. Histological sections employing Nissl´ staining from a healthy control mouse (B) and a resuscitated animal five days after CA/CPR (D) showing the hippocampus (left panels) and a magnification of the hippocampal CA1/CA-2 region (right panels). Following CA/CPR, pyramidal cells of the CA-1 region show signs of neuronal cell apoptosis (right panel, D). (C) and (D) are findings in the same animal that suggest an excellent correlation between NMR and histological examinations. (TIF) [file pone.0074944.s001.tif]

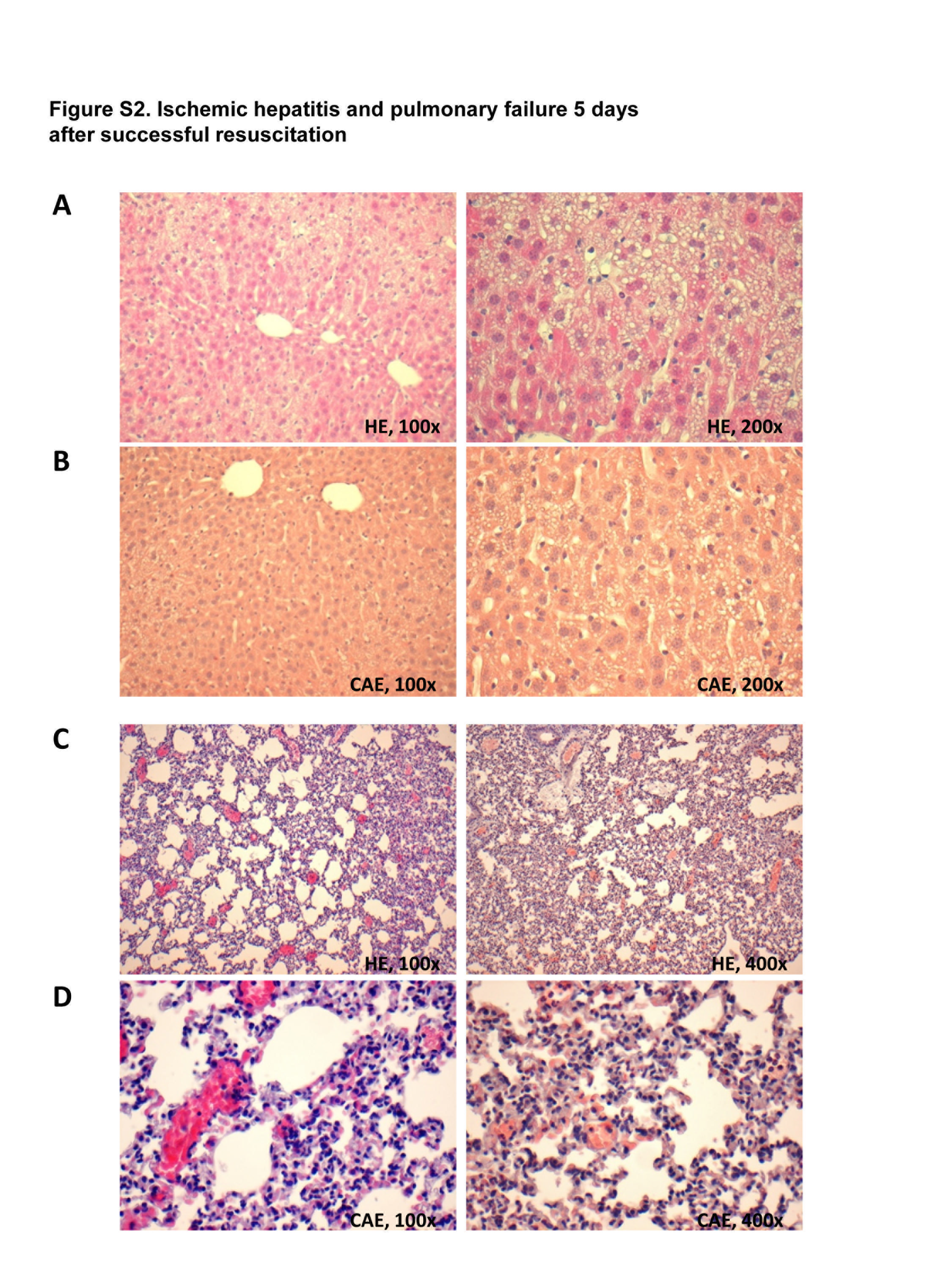

Supplement: Figure S2 — Ischemic hepatitis and pulmonary failure 5 days after successful resuscitation. Histological sections of the liver of a WT mouse five days after CA/CPR, signs of ischemic hepatitis and apoptotic cells are visible in hematoxylin eosin (HE) (A) and leukocyte infiltration in chloroacetate esterase (CAE) staining (B). C Histological sections of a WT mouse lung five days after CA/CPR exhibiting pulmonary oedema and alveolar membrane thickening in HE and D leukocyte infiltration in CAE staining. 100x and 200x indicate magnification. (TIF) [file pone.0074944.s002.tif]
