# Supplementary material for: Impact of Toll-Like Receptor 2 Deficiency on Survival and Neurological Function after Cardiac Arrest: A Murine Model of Cardiopulmonary Resuscitation
Source: PLoS One. 2013 Sep 16;8(9):e74944. doi: 10.1371/journal.pone.0074944 (PMC3774715; doi:10.1371/journal.pone.0074944)
Supplement: Checklist S1 — ARRIVE guidelines. Checklist for the experimental setup based on the ARRIVE-guidelines (Animals in Research: Reporting in vivo Experiments) Introduction: “Impact of Toll-like receptor 2 deficiency on survival and neurological function after cardiac arrest: A murine model of cardiopulmonary resuscitation”. (DOCX) [file pone.0074944.s003.docx]

**Checklist S1:**

| **Title** | Provide as accurate and concise a description of the content of the article as possible | Toll-like receptor2 deficiency and inhibition improves survival and neurological function in the post-resuscitation phase in a murine model of cardiopulmonary resuscitation. | **✓** |
| --- | --- | --- | --- |
| **Abstract** | Provide and accurate summary of the background, research objectives, including details of the species or strain of animal used, key methods, principal findings and conclusion of the study |  | **✓** |
| **Introduction** |  |  |  |
| Background | 1. Include sufficient scientific background (including relevant references to previous work) to understand the motivation and context for the study, and explain the experimental approach and rationale. | Menzebach A, Bergt S, von Waldthausen P, Dinu C, Nöldge-Schomburg G, Vollmar B. A comprehensive study of survival, tissue damage, and neurological dysfunction in a murine model of cardiopulmonary resuscitation after potassium-induced cardiac arrest. Shock 2010;33:189-196  Lorne E, Dupont H, Abraham E. Toll-like receptors 2 and 4: initiators of non-septic inflammation in critical care medicine? Intensive Care Med 2010;36:1826-1835  Arslan F, Smeets MB, O'Neill LA, Keogh B, McGuirk P, Timmers L, Tersteeg C, Hoefer IE, Doevendans PA, Pasterkamp G, de Kleijn DP. Myocardial ischemia/reperfusion injury is mediated by leukocytic toll-like receptor-2 and reduced by systemic administration of a novel anti-toll-like receptor-2 antibody. Circulation 2010;121:80-90  Farrar CA, Keogh B, McCormack W, O'Shaughnessy A, Parker A, Reilly M, Sacks SH. Inhibition of TLR2 promotes graft function in a murine model of renal transplant ischemia-reperfusion injury. FASEB J 2012;26:799-807  Ziegler G, Freyer D, Harhausen D, Khojasteh U, Nietfeld W, Trendelenburg G. Blocking TLR2 in vivo protects against accumulation of inflammatory cells and neuronal injury in experimental stroke. J Cereb Blood Flow Metab 2011;31:757-766 | **✓** |
|  | 1. Explain how and why the animal species and model being used can address the scientific objective and, where appropriate, the study´s relevance to human biology. | We used a murine model to simulate the pathological conditions of cardiac arrest and the post resuscitation disease. We used mice as laboratory animals in order to use genetically altered animals as proof of principle and afterwards to use a pharmacological approach as a therapeutic option.  The present study is considered to be basic science showing that TLR2 inhibition offers protective effects after CA/CPR. However, a direct translation of the present results into human treatment of diseases should be done with great cautions. |  |
| Objectives | Clearly describe the primary and any secondary objectives of the study, or specific hypotheses being tested. | In a murine model of CA/CPR we aimed to investigate the effects of TLR2 deficiency or treatment with TLR2 blocking antibodies on survival and neurological function in the 28-day post-resuscitation phase. We hypothesised that inhibition of TLR2 pathways improves survival and neurofunctional regeneration after CA/CPR. | **✓** |
| **Methods** |  |  |  |
| Ethical statement | Indicate the nature of the ethical review permission, relevant licences (e.g. Animal [Scientific Procedure] Act 1986), and national or international guidelines for the care and use of animals, that cover the research. | All animal experiments were approved by the governmental ethical board for animal research (LALLF M-V/TSD/7221.3-1.1-073/10) and in accordance with the European Communities Council Directive of November 24th, 1986 (86/609/EEC). | **✓** |
| Study design | 1. The number of experimental and control groups. | One control group and two interventional groups:  WT (control group): 35 female wild-type mice (WT, C57BL/6J)  TLR2-/- group: 35 female B6.129-Tlr2tm1Kir/J mice  WT+T2.5: 17 female WT mice (WT, C57BL/6J) subjected to intravenous treatment with a TLR2 inhibiting antibody (T2.5) | **✓** |
|  | 1. Any steps taken to minimise the effects of subjective bias when allocating animals to treatment (e.g. randomisation procedure) and when assessing results (e.g. if done, describe who was blinded and when). | The trial was randomized, placebo controlled and investigator blinded.  Randomization: Randomization was performed by drawing by lot. This process was blinded only for the WT and TLR2-/-, both groups receiving vehicle solution at CPR. The WT+2.5 group was not randomized. Due to identical phenotypes of all study groups and the injection of the TLR2 blocking antibody randomization in a blinded fashion was not possible.  Bodyweight, Neuro score, RotaRod and Water Maze tests were performed by a blinded observer (A. Güter). Due to identical phenotypes blinding was possible. | **✓** |
|  | 1. The experimental unit (e.g. a single animal, group or cage of animals). | 5 mice were housed in one cage. Mice were returned to cages after recovering from CPR and anaesthesia. | **✓** |
| Experimental procedures | 1. How (e.g. drug formulation and dose, site and route of administration, anaesthesia and analgesia used [including monitoring], surgical procedure, method of euthanasia). Provide details of any specialist equipment used, including supplier(s). | - anaesthesia for the experiments is performed by intra peritoneal injection  - drug formulations, dosages and suppliers were adapted from the literature  - all interventional medication (TLR2 anti-body) was applied by iv-injection (central catheter)  - all monitoring is named and information about manufacturers are given  - all surgical procedures are described in detail.  - all specialist equipment is named and information about manufacturers are given  - the resuscitation device was self-build by modifying a sewing machine | **✓** |
|  | 1. When (e.g. time of day) | - all experiments were performed in the morning at the same time | **✓** |
|  | 1. Where (e.g. home cage, laboratory, water maze). | - all neurotesting, laboratory work and housing of animals were done in the Institute for Experimental Surgery, Rostock University, Rostock, Germany | **✓** |
|  | 1. Why (e.g. rationale for choice of specific anaesthetic, route of administration, drug dose used). | - intraperitoneal injection aneasthetics is standard route of inducing anaesthesia  - drug formulations, dosages and suppliers were adapted from the literature  - the TLR2 blocking antibody was injected intravenously to ensure 100% bioavailability | **✓** |
| Experimental animals | 1. Provide details of the animals used, including species, strain, sex, developmental stage (e.g. mean or median age plus age range) and weight (e.g. mean or median weight plus weight range) | control group: 35 female wild-type mice (WT, C57BL/6J) (body weight [BW] 20–24 g, age 12–16 weeks)  TLR2-/- group: 35 female B6.129-Tlr2tm1Kir/J mice (BW 20–24 g, age 12–16 weeks)  WT+T2.5: 17 female WT mice (WT, C57BL/6J) [BW] 20–24 g, age 12–16 weeks), subjected to intravenous treatment with a TLR2 inhibiting antibody (T2.5) | **✓** |
|  | 1. Provide further relevant information such as the source of animals, international strain nomenclature, genetic modification status (e.g. knock-out or transgenic), genotype, health/immune status, drug or test naïve, previous procedures, etc. | All animals were purchased from Charles River, Germany.  All mice had the same phenotype. The functional deletion of the TLR2 receptor was proven by Western blotting.  All mice were healthy at the beginning of the experiments (health certificates) and did not participate in any other study and were not subjected to any pretreatment. | **✓** |
| Housing and husbandry | 1. Housing (type of facility e.g. specific pathogen free [SPF]; type of cage or housing; bedding material; number of cage companions; tank shape and material etc. for fish). | Mice were housed in a temperature-controlled room (temperature 22°C, humidity 45%) under a 12-h dark light cycle. Mice were housed in cages with 5 mice each. All animals had free access to water and standard laboratory food. | **✓** |
|  | 1. Husbandry conditions (e.g. breeding program, light/dark cycle, temperature, quality of water etc. for fish, type of food, access to food and water, environmental enrichment). | Mice were housed in a temperature-controlled room (temperature 22°C, humidity 45%) under a 12-h dark light cycle. Mice were housed in cages with 5 mice each. All animals had free access to water and standard laboratory food. | **✓** |
|  | 1. Welfare-related assessments and interventions that were carried out prior to, during, or after the experiment | All mice were closely monitored for their wellbeing:  - measurement of bodyweight twice daily  - observation of wellbeing and survival during the day (8 am to 8 pm) every 2 hours; at night every 4 hours. Physiological parameters: breathing pattern; spontaneous movement; ability to ingest food; grooming  - daily neurotesting  - daily RotaRod testing  - daily Water Maze testing | **✓** |
| Sample size | 1. Specify the total number of animals used in each experiment, and the number of animals in each experimental group. | 28 day observation experiments:  WT (control group): 35 mice; 4 mice were excluded  TLR2-/-: 35 mice; 5 mice were excluded  WT+2.5: 17 mice; 2 mice were excluded  Exclusion criteria: - problems during surgical preparation or CPR (i.e. massive blood loss, dislocation of central venous catheter during resuscitation procedure, accidental extubation)  8 hours experiments:  Control group: 6 mice  TLR2-/-: 6 mice  WT+2.5: 6 mice | **✓** |
|  | 1. Explain how the number of animals was arrived at. Provide details of any sample size calculation used. | During the phase of establishing the CA/CPR murine model we observed a mortality of 50% in the control group after an observation period of 28 days.  Assuming a protective effect of a therapy of 15-30% and a distribution of results of 10% there has to be at least a sample size of 30 mice/ group. Assuming a loss of animals of 7.5 % - a value derived from the establishing phase of our model – a sample size of 35 animals per group is necessary.  However, due to high costs of the TLR2 antibody the experiments of the WT+T2.5 group were stopped after 17 mice after observing a high degree of protection. | **✓** |
|  | 1. Indicate the number of independent replications of each experiment, if relevant. |  |  |
| Allocating animals to  Experimental groups | 1. Give full details of how animals were allocated to experimental groups, including randomisation or matching if done. | Randomization: Randomization was performed by drawing by lot. This process was blinded only for the WT and TLR2-/-, both groups receiving vehicle solution at CPR. The WT+2.5 group was not randomized. Due to identical phenotypes of all study groups and the injection of the TLR2 blocking antibody randomization in a blinded fashion was not possible.  Bodyweight, Neuro score, RotaRod and Water Maze tests were performed by a blinded observer (A. Güter). Due to identical phenotypes blinding was possible. | **✓** |
|  | 1. Describe the order in which the animals in the different experimental groups were treated and assessed. | Allocation of mice to experimental groups was performed randomly (drawing by lot). All animals were treated and assessed in an identical fashion. |  |
| Experimental outcomes | Clearly define the primary and secondary experimental outcomes assessed (e.g. cell death, molecular markers, behavioural changes). | Survival over 28 days  neurological function assessed by:  - Neuro score  - RotaRod test  - Water Maze test  Inflammatory response by:  - corticosterone  - IL-6  - IL-1β | **✓** |
| Statistical methods | 1. Provide details of the statistical methods used for each analysis. | Data are expressed as median with [25–75] percentiles. Differences between groups were assessed by means of Mann-Whitney rank sum test. Survival data for Kaplan-Meier curves were tested with the log-rank test. Statistical analysis was performed employing SigmaPlot 10 (Jandel Corporation, San Rafael, CA, USA) and statistical significance was defined as p<0.05. | **✓** |
|  | 1. Specify the unit of analysis for each dataset (e.g. single animal, group of animals, single neuron). | Group of animals (experimental groups: WT, TLR2-/-, WT+2.5) | **✓** |
|  | 1. Describe any methods used to assess whether the data met the assumptions of the statistical approach. | Because of small sample sizes (in comparison to population) not normal distribution was assumed and therefore non-parametric testing was used. | **✓** |
| **Results** |  |  |  |
| Baseline data | For each experimental group, report relevant characteristics and health status of animals (e.g. weight, microbiological status, and drug or test naïve) prior to treatment or testing. (This information can often be tabulated). | All experimental animals were healthy (health certificate) at baseline, had no neurological deficits and were naïve to drugs or medication prior to the experiments. | **✓** |
| Numbers analysed | 1. Report the number of animals in each group included in each analysis. Report absolute numbers (e.g. 10/20, not 50%). | 28 day observation experiments/survival:  WT (control group): 35 mice; 4 mice were excluded 🡪 31 animals were analysed; 15 mice dyed 🡪 approximately 51% survival  TLR2-/-: 35 mice; 5 mice were excluded 🡪 30 mice were analysed; 7 mice dyed 🡪 approximately 77% survival  WT+2.5: 17 mice; 2 mice were excluded 🡪 15 mice were analysed; 3 mice dyed 🡪 80% survival  Exclusion criteria: - problems during surgical preparation or CPR (i.e. massive blood loss, dislocation of central venous catheter during resuscitation procedure, accidental extubation)  8 hours experiments:  Control group: 6 mice  TLR2-/-: 6 mice  WT+2.5: 6 mice  All 18 mice were sacrificed in order to obtain serum for laboratory analysis. | **✓** |
|  | 1. If any animals or data were not included in the analysis, explain why. | In the 28 day observation experiments 11 mice had to be excluded due to death not related to CA/CPR.  Exclusion criteria: - problems during surgical preparation or CPR (i.e. massive blood loss, dislocation of central venous catheter during resuscitation procedure, accidental extubation) | **✓** |
| Outcomes and estimation | Report the results for each analysis carried out, with a measure of precision (e.g. standard error or confidence interval). | The majority of results is expressed as median with 25/75 quartiles. | **✓** |
| Adverse events | 1. Give details of all important adverse events in each experimental group. | There were no adverse events. |  |
|  | 1. Describe any modifications to the experimental protocols made to reduce adverse events. |  |  |
| **Discussion** |  |  |  |
| Interpretation / Scientific implications | 1. Interpret the results, taking into account the study objectives and hypotheses, current theory and other relevant studies in the literature. | Done | **✓** |
|  | 1. Comment on the study limitations including any potential sources of bias, any limitations of the animal model, and the imprecision associated with the results. | Due to limitation of words (3000 words) this section was covered short. | **✓** |
|  | 1. Describe any implications of your experimental methods or findings for the replacement, refinement or reduction (the 3Rs) of the use of animals in research. | We use a highly standardized (by means of frequency of compressions and depth of compressions) murine model of cardiac arrest (CA) and cardiopulmonary resuscitation (CPR). The development of this model lasted for 3 years. It is adapted to a clinical setting (i.e. treatment after CA, long term observation, focus on neuro-functional impairment besides survival. The pathological state of cardiac arrest, the therapy with cardiopulmonary resuscitation and the observation of the course of post resuscitation disease cannot be simulated by computer or transferred into a cellular approach. Therefore, due to the complexity of pathological mechanisms the transfer in an animal model is necessary and reasonable. | **✓** |
| Generalis-ability / Translation | Comment on whether, and how, the findings of this study are likely to translate to other species or systems, including any relevance to human biology. | The present study is considered to be basic science showing that TLR2 inhibition offers protective effects after CA/CPR. This finding is congruent with results of other research groups who investigate reperfusion injury in other organs and tissues. A translation into other species seems reasonable, however, a direct translation of the present results into human treatment of diseases should be done with great cautions. | **✓** |
| Funding | List all funding sources (including grant number) and the role of the funder(s) in the study. | This study was not funded by any institution or company. | **✓** |


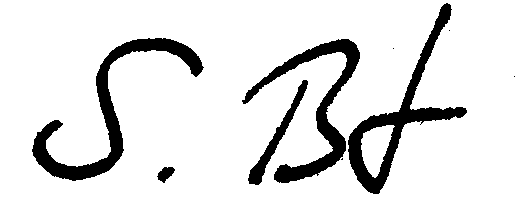


Rostock, den 23.07.2013 Dr. Stefan Bergt
